# Isolation and Characterization of Bacteria from Ancient Siberian Permafrost Sediment

**DOI:** 10.3390/biology2010085

**Published:** 2013-01-10

**Authors:** De-Chao Zhang, Anatoli Brouchkov, Gennady Griva, Franz Schinner, Rosa Margesin

**Affiliations:** 1 Institute of Microbiology, University of Innsbruck, Technikerstrasse 25, A-6020 Innsbruck, Austria; E-Mails: zhangdechao@qdio.ac.cn (D.-C.Z.); franz.schinner@uibk.ac.at (F.S.); 2 Faculty of Geology, Lomonosov Moscow State University, GSP-1,1 Leninskiye Gory, Moscow 119991, Russia; E-Mail: brouchkov@hotmail.com; 3 Tyumen Scientific Center Siberian Branch of Russian Academy of Science, 86 Malygina, Tyumen 625000, Russia; E-Mail: grivag@mail.ru

**Keywords:** permafrost, ancient, Neogene, sediment, Siberia, microorganisms, *Arthrobacter*, *Glaciimonas*, *Subtercola*

## Abstract

In this study, we isolated and characterized bacterial strains from ancient (Neogene) permafrost sediment that was permanently frozen for 3.5 million years. The sampling site was located at Mammoth Mountain in the Aldan river valley in Central Yakutia in Eastern Siberia. Analysis of phospolipid fatty acids (PLFA) demonstrated the dominance of bacteria over fungi; the analysis of fatty acids specific for Gram-positive and Gram-negative bacteria revealed an approximately twofold higher amount of Gram-negative bacteria compared to Gram-positive bacteria. Direct microbial counts after natural permafrost enrichment showed the presence of (4.7 ± 1.5) × 10^8^ cells g^−1^ sediment dry mass. Viable heterotrophic bacteria were found at 0 °C, 10 °C and 25 °C, but not at 37 °C. Spore-forming bacteria were not detected. Numbers of viable fungi were low and were only detected at 0 °C and 10 °C. Selected culturable bacterial isolates were identified as representatives of *Arthrobacter phenanthrenivorans*, *Subtercola frigoramans* and *Glaciimonas immobilis*. Representatives of each of these species were characterized with regard to their growth temperature range, their ability to grow on different media, to produce enzymes, to grow in the presence of NaCl, antibiotics, and heavy metals, and to degrade hydrocarbons. All strains could grow at −5 °C; the upper temperature limit for growth in liquid culture was 25 °C or 30 °C. Sensitivity to rich media, antibiotics, heavy metals, and salt increased when temperature decreased (20 °C > 10 °C > 1 °C). In spite of the ligninolytic activity of some strains, no biodegradation activity was detected.

## 1. Introduction

Permafrost is one of the most extreme environments on earth and covers more than 20% of the earth’s land surface; it has been defined as lithosphere material (soil, sediment or rock) that is permanently exposed to temperatures ≤0 °C and remains frozen for at least two consecutive years, and can extend down to more than 1,500 m in the subsurface [1]. Regions with permafrost occur at high latitudes, but also at high elevations; a significant part of the global permafrost is represented by mountains [2].

The microbial long-term survival in permafrost has been questioned; however, there is evidence that bacteria are able to survive in 500,000-year-old permafrost [3]. Considerable abundance and diversity of microorganisms, including bacteria, archaea, phototrophic cyanobacteria and green algae, fungi and protozoa, are present in permafrost [4,5,6]. The characteristics of these microorganisms reflect the unique and extreme conditions of the permafrost environment. Permafrost soils may contain up to 20% or more unfrozen water in the form of salt solutions with a low water activity (a_w_ = 0.8–0.85) [7]. Microorganisms in this environment have additionally to thrive under permanently frozen conditions, oligotrophic conditions, complete darkness, constant gamma radiation and extremely low rates of nutrient and metabolite transfer [4,5]. Substantial growth and metabolic activity (respiration and biosynthesis) of permafrost microorganisms at temperatures down to −20 °C and even −35 °C have been demonstrated [8,9,10].

Relict microorganisms from ancient permafrost are not only of interest from an ecological point of view, recent studies pointed to their significance as objects of gerontology. *Bacillus* sp. isolated from permafrost sands of the Mammoth Mountain in Central Yakutia was characterized by an extraordinary viability (about 3.5 million years old) and enhanced longevity, immunity and resistance to heat shock and UV irradiation in *Drosophila melanogaster* and mice [11,12,13,14]; probiotic activity by a *Bacillus* sp. strain isolated from the same sample has been recently reported [15].

Frozen soils consisting of mineral particles and ice of different ages contain live microorganisms [16]. It has been shown that microbial cells, even showing features of aging [17,18], are able to live or stay viable for a long time. Despite the fact that it is unknown whether these cells are individually surviving or growing, *Bacillus anthracis* remains viable for about 10^5^ years [19]. Colonies of bacteria from amber have been reported to survive for 40 million or more years [20].

Viability of bacteria below 0 °C has been investigated [21]. Unfrozen water, held tightly by electrochemical forces onto the surfaces of mineral particles, occurs even in hard-frozen permafrost. Bacterial cells are not frozen at temperatures of −2 °C and −4 °C [22,23]. The thin liquid layers provide a route for water flow, carrying solutes and small particles, possibly nutrients or metabolites, but movement is extremely slow. A bacterium of greater size (0.3–1.4 µm) than the thickness of the water layer (0.01–0.1 µm at temperatures of −2 °C and −4 °C) is unlikely to move, at least in ice [2]. Therefore, microorganisms trapped among mineral particles and ice in permafrost have been isolated [16]. In some cases, their age can be proved by geological conditions, the history of freezing, and radioisotope dating [21].

The nature of extreme longevity of permafrost microorganisms has no comprehensive explanation. Cell structures are far from being stable [24]. The genome is subject to destruction, and the reparation mechanisms of the majority of organisms are not effective enough to prevent accumulation of damages [25]. The half-life of cytosine does not exceed a few hundred years [26]. Ancient DNA of mummies, mammoths, insects in amber and other organisms appears destroyed [20,27,28].

Microorganisms in permafrost have been studied by culture-dependent and culture-independent methods [4,5,6]. Microbial abundance is often based on culture-based methods. However, culturable cells may only represent less than 1% of the total microbial community in an environment [29] and numerous bacteria enter a viable but non-culturable (VBNC) state in response to environmental stress [30]. Therefore, culture-independent, molecular assays, such as profiling soil DNA, rRNA, or phospholipid fatty acids, are increasingly used in environmental microbiology. Direct recovery of bacterial 16S rDNA theoretically represents the entire microbial population from environmental samples [31]. However, molecular methods also have their limitations, such as variable efficiency of lysis and DNA extraction, and differential amplification of target genes [32]. Only through isolation can microorganisms be fully characterized at the physiological and functional level. Although major advances have been made in the last decade, our knowledge on the genetics, biochemistry and ecology of microorganisms in permafrost is still limited.

In this study, we investigated the culturable heterotrophic microbial population in ancient (Neogene) permafrost collected from one of the oldest permafrost areas on earth, located in Siberia and permanently frozen for 3.5 million years. We analyzed the bacterial and fungal population by using a combination of culture-dependent and culture-independent techniques. Selected bacterial isolates were characterized with regard to their growth characteristics, their ability to grow on different media, to produce enzymes and to degrade hydrocarbons, and their sensitivity to NaCl, antibiotics, and heavy metals.

## 2. Materials and Methods

### 2.1. Sampling Site

The sampling site was located at Mammoth Mountain in the Aldan river valley in Central Yakutia in Eastern Siberia. The site is an exposure located on the left bank of the Aldan river, 325 km upstream from the mouth of the River Lena (N62°56' E134°0.1'). The exposure is a consequence of recent river erosion of a few cm, up to 0.7 m per year. Prior to the erosion, the sampling site would have been considerably deeper.

Annual mean temperature of the deposits is presently about −4 °C near the surface; the temperature is constantly below 0 °C. Alluvial deposits consisting of fine-grained sands and aleurolites with interlayers of plant remains (trunks, branches, leaves) are exposed. The systematic composition of seeds, pollen and leafs is related to Middle Miocene [33], about 11–16 million years ago. This is the northernmost part of the known Eurasian localities of Neogenic leaf and trunk remains of *Salix*, *Populus*, *Alnus* and other species. The deposits in the area became frozen at least 1.8–1.9 million years ago [34], and probably earlier than 3.5 million years ago, and never thawed until now because of the cold climate of the Pleistocene [2]. Recent studies showed that an intensive cooling began there in Late Pliocene 3–3.5 million years ago. The temperature was estimated as ranging from −12 °C to −32 °C in January and from about +12 °C to +15.6 °C in July [35], thus the age of the permafrost at Mammoth Mountain likely reaches up to 3.5 million years. Geological data indicated the absence of thawing of deposited sediment for millions of years, which assures the ancient age of the sample [15].

**Figure 1:**
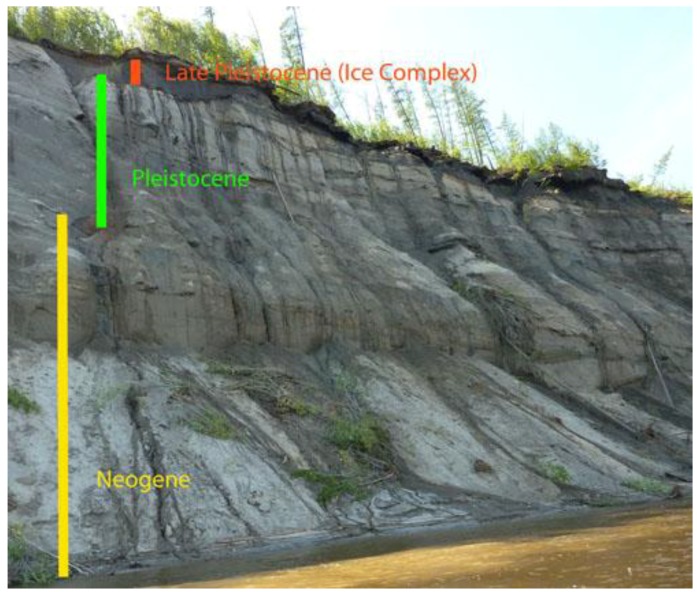
Sampling site at Mammoth Mountain in the Aldan river valley (Central Yakutia, Eastern Siberia). Formations of Late Pleistocene (Ice Complex) (red), Pleistocene (green) and Neogene (yellow) are visible.

**Figure 2:**
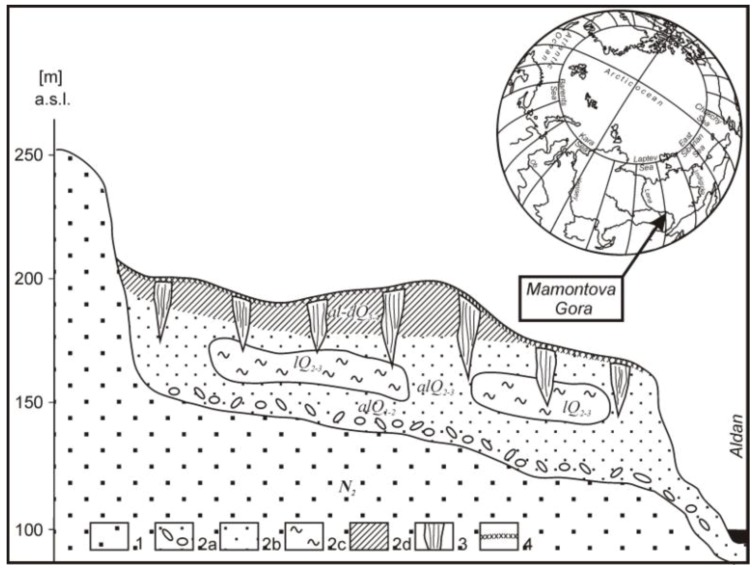
Profile of the exposure of the Mammoth Mountain: **1**, Neogene sands; **2**, Pleistocene sediment: **a**, pebbles in the ferrous sands; **b**, sands; **c**, lacustrine silt; **d**, silt; **3**, ice wedge; **4**, active layer (after data from Markov [36]).

**Figure 3:**
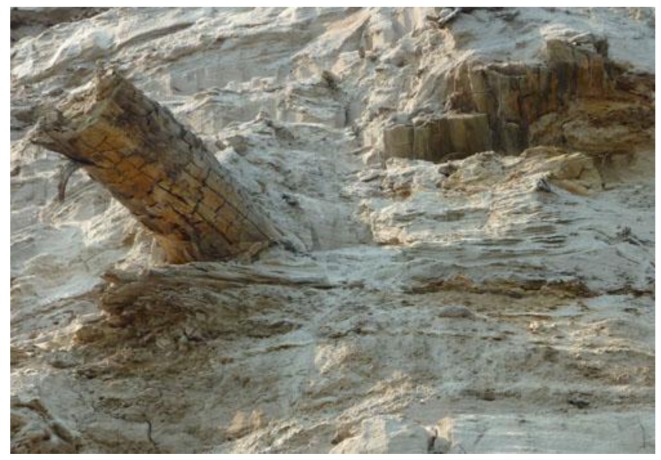
A frozen trunk slightly decomposed was found about 15 m above the river level in the Middle Miocene (Neogene) deposits (3.5 million years old).

The exposure has three visible major ancient layers that can be attributed to Late Pleistocene (about 15,000–40,000 years old Ice Complex), Middle and Early Pleistocene (sands and clay; 0.1–1 million years old, frozen at the time of formation) and Neogene (Miocene and Pliocene), mostly sand formations, frozen probably at the end of Neogene about 3.5 million years ago (Figure 1 and Figure 2). A slightly decomposed frozen trunk (Figure 3) was found about 15 m above river level in the Middle Miocene deposits. The topsoil on the exposure, an active layer of about 0.9–1 m, is of modern age and consisted of (acidic) raw humus on frozen siliceous sand and silt and was covered with vegetation that consisted of birch, alder, conifers (spruce, larch and pine tree) and shrubs.

### 2.2. Sampling

**Figure 4:**
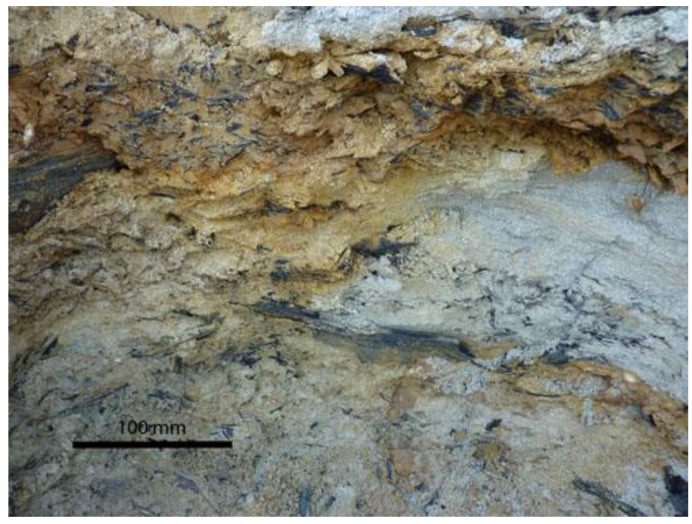
Permafrost immediately before sampling. Neogenic deposits consisting of fine-grained sands and aleurolites with interlayers of plant remains characterize the sediment at the site.

Samples were collected in July 2009 at an altitude of 83 m above sea level, exposition north, and at a depth of 1.5 m from the surface of the Neogene formation (Figure 4). A deep hole of approximately 100 cm was horizontally dug into the frozen Neogene horizon. After sterilizing the surface of this sampling hole by flame, pieces of frozen sediment (icy sand) were collected from a horizontal depth of 75–100 cm, cleaved with a sterilized axe, and collected in sterile 50 mL vials by using sterile spatulas. The mean temperature of the icy sand at the time of sampling was −4 °C.

Samples were immediately embedded in frozen natural permafrost material, then stored in a cryogenic mixture of NaCl and water to keep the material constantly frozen. The samples were kept frozen during transport from Yakutia to the laboratory in Innsbruck where samples were stored at −20 °C. Thus, the collected material was constantly kept frozen and never subjected to thawing. A composite sample was produced under sterile conditions immediately before analysis.

### 2.3. Enrichment of Microorganisms

The composite sample was kept for one month at 0 °C for natural permafrost enrichment (NPE) [37]. Afterwards, a number of analyses (physical and chemical soil properties, PLFA, direct and viable microbial counts) were performed.

After NPE, a liquid enrichment (LE) culture was produced by preparing a 1:20 dilution of the NPE with 1/10 strength R2A broth. This enrichment culture was kept at 1 °C on a shaker at 100 rpm. After two and four weeks, samples were analyzed again for direct and viable microbial counts.

### 2.4. Physical and Chemical Sediment Properties

Dry mass content was determined from mass loss after 24 h at 105 °C. Soil organic matter (SOM) was determined from loss on ignition (LOI) after heating dried soil for 3 h at 430 °C [38]. Soil pH was determined in 10 mM CaCl_2_ [39]. Contents of nitrate, nitrite and phosphorus were determined spectrophotometrically [39].

### 2.5. Phospholipid Fatty Acids (PLFA)

Phospholipids were determined after NPE as described [40] and were extracted from 6 g (fresh mass) of sediment, fractionated and quantified using the procedures described [41,42]. Separated fatty acid methyl-esters were identified using gas chromatography and a flame ionization detector. Fatty acid nomenclature was used as described [41]. The fatty acids i15:0, a15:0, 15:0, i16:0, 16:1ω7c, 17:0, i17:0, cy17:0, 18:1ω7c and cy19:0 were chosen to represent bacterial biomass (bacterial PLFA), and 18:2ω6,9c (fungal PLFA) was taken to indicate fungal biomass [43,44]. The ratio of bacterial PLFA to fungal PLFA was calculated to indicate shifts in the ratio between bacterial and fungal biomass. The Gram-positive specific fatty acids i15:0, a15:0, i16:0 and i17:0 and the Gram-negative specific fatty acids cy17:0 and cy19:0 [45] were taken as a measure of the ratio between Gram-positive and Gram-negative bacteria. The fatty acid 20:5ω3c was used as an indicator for soil algae [46]. PLFA concentrations (nmol g^−1^ sediment) were calculated on a dry mass basis and were determined with three replicates.

### 2.6. Direct Microbial Counts

Total microbial counts were determined by using acridine orange staining and Calcofluor-white staining and epifluorescence microscopy [47,48]. After NPE, 1 mL 10^−2^ diluted sediment extract (the same that was also used for the determination of viable microbial counts) was stained with 1 mL of 0.01% acridine orange or with 1 mL of Calcofluor white M2R (15 µg mL^−1^) for 3 min. To remove excess staining, the stained suspension was filtered through a 0.4 µm pore size filter (Millipore HTBP02500 Isopore black) held on a 25 mm diameter filter holder. The filter was air dried, cleared in immersion oil and covered by a cover glass. Slides were examined with a Nikon Microphot-SA epifluorescent microscope equipped with a high intensity mercury light source. A Nikon B-2A filter cube was used for examination of acridine orange stained slides. Ten randomly-chosen fields of view were photographed with an 8-bit digital color camera (Nikon Digital sight DS U1) and cells were counted.

### 2.7. Enumeration of Culturable Heterotrophic Aerobic Sediment Microorganisms

Culturable microorganisms in the sediment sample were enumerated with three replicates by the plate-count method for viable cells. Pre-chilled glassware and solutions were used. Sediment suspensions were prepared by shaking sediment after NPE (10 g fresh mass) for 15 min at 150 rpm with 90 mL of ice-cold sodium pyrophosphate solution (0.28%). Dilutions of this sediment suspension prepared in ice-cold pyrophosphate solution were surface spread onto agar plates. Similarly, the liquid enrichment culture was diluted in ice-cold pyrophosphate solution and surface spread onto agar plates.

R2A agar and 1/10 strength R2A agar (prepared as R2A broth diluted 1:10 with sterile distilled water and supplemented with agar) were used to determine numbers of viable heterotrophic bacteria. To determine numbers of spore-forming bacteria, the dilutions of the sediment suspension were kept for 15 min at 80 °C in a water bath and afterwards spread on R2A and 1/10 strength R2A agar. Saboraud agar, 1/10 strength Saboraud agar, and malt extract agar (each of these media was supplemented with chlorampenicol (100 µg mL^−1^) and tetracyclin (100 µg mL^−1^) to inhibit bacterial growth) were used to determine numbers of viable fungi. Sterile controls were incubated under the same conditions as inoculated plates.

All plates were incubated at −5 °C, 0 °C, 10 °C, 25 °C and 37 °C. Colonies were incubated up to 42 days (−5 °C and 0 °C), 28 days (25 °C), 5–14 days (25 °C) or 7 days (37 °C) until no growth of new colonies was detected. Colony-forming units (CFU) were calculated on a sediment dry mass basis.

### 2.8. Phylogenetic Analysis of Culturable Bacteria and Restriction Fragment Length Polymorphism (RFLP)

Genomic DNA of 32 culturable bacterial strains (collected from plates incubated at 0 °C) differing in phenotypic characteristics (colony morphology, pigmentation, growth characteristics) was extracted using the UltraClean Microbial DNA isolation kit (Mo Bio Laboratories). The 16S rRNA genes were amplified as described earlier [49].

Restriction fragment length polymorphism (RFLP) was carried out as described [50]. Amplified 16S rRNA genes were restricted using the enzymes RsaI and HhaI (Invitrogen) at 37 °C overnight. Restriction digests were analyzed by agarose gel electrophoresis (2% agarose, 0.5× TBE buffer). Unique restriction patterns were identified visually and two representatives of each restriction pattern were used as a template for 16S rRNA gene sequencing. Sequencing reactions were carried out by Eurofins MWG Operon (Ebersberg, Germany). The 16S rRNA gene sequences were submitted for comparison and identification to the GenBank databases using the NCBI Blastn algorithm and to the EMBL databases using the Fasta algorithm.

### 2.9. Characterization of Culturable Bacteria

#### 2.9.1. Growth Temperature Range

Growth at −5, 1, 5, 10, 15, 20, 25, 30 and 35 °C was assessed on R2A agar and in R2A broth at 150 rpm (except for cultures at −5 °C where shaking was not possible), using two replicates per strain and temperature. Growth on agar plates was regularly monitored up to an incubation time of 28 days; growth in liquid cultures was monitored by measuring regularly OD600.

#### 2.9.2. Growth on Different Media

Growth on different media was assessed on 1/10 strength R2A agar, R2A agar, nutrient agar (NA, 0.5% peptone, 0.3% meat extract, 1.5% agar; pH 7), TSA (trypticase soy agar; 1.5% casein peptone, 0.5% soy peptone, 0.5% sodium chloride, 1.5% agar; pH 7) and LB agar (Luria Bertani agar; 1% tryptone, 0.5% yeast extract, 0.5% NaCl). Plates were incubated at 1 °C, 10 °C and 20 °C and growth was monitored up to an incubation time of 21 days.

#### 2.9.3. Facultatively Anaerobic Growth

Facultative growth under anaerobic conditions was determined as described [51] on R2A agar, on half-concentrated nutrient agar and on nutrient agar supplemented with 10 mM KNO_3_ after incubation at 1 °C, 10 °C and 20 °C in an anaerobic jar (containing Anaerocult A (Merck) to produce anaerobic conditions).

#### 2.9.4. Salt Tolerance

Growth in the presence of various salt concentrations was determined on R2A agar supplemented with 0, 1, 2, 3, 5, 7 and 10% (w/v) NaCl. Two replicates per strain and NaCl concentration were tested. Plates were incubated at 1 °C, 10 °C and 20 °C and growth was monitored up to an incubation time of 21 days.

#### 2.9.5. Resistance to Antibiotics

Susceptibility to antibiotics was determined on R2A agar supplemented with penicillin, ampicillin, kanamycin, streptomycin, rifampicin, tetracyclin, chloramphenicol and cyclosporin. Two concentrations (20 and 100 µg mL^−1^) were tested for each antibiotic. Growth was tested with two replicates per strain, antibiotic and temperature at 1 °C, 10 °C and 20 °C. Growth was regularly monitored up to an incubation time of 21 days. Strain N1-17 was additionally tested for its susceptibility to antibiotics (20 µg mL^−1^) in R2A broth at 1 °C, 10 °C and 20 °C; growth in liquid cultures was monitored by measuring regularly OD600.

#### 2.9.6. Resistance to Heavy Metals

Resistance to heavy metals was determined on a mineral salts medium in order to avoid complexation of heavy metals in a complex medium [52]. The used medium was pH-neutral and Tris-buffered [53] and contained 0.1% glucose and 0.1% gluconate as carbon sources, and 1.5% purified agar. The medium was supplemented with the heavy metals Zn^2+^ (1, 3, 5 mM; supplied as Zn(NO_3_)_2_ × 6H_2_O), Pb^2+^ (1, 3, 5 mM; supplied as Pb(NO_3_)_2_) or Cu^2+^ (0.1, 1, 2, 3 mM; supplied as CuSO_4_). All metals were provided in a soluble, bioavailable form. Plates were incubated up to 21 days at 1, 10 and 20 °C.

#### 2.9.7. Biodegradation of Hydrocarbons

Biodegradation of hydrocarbons was tested as described [54] on mineral medium agar plates amended with the following hydrocarbons as the sole carbon source: n-hexadecane (2,000 mg L^−1^), diesel oil (2,500 mg L^−1^), phenol (2.5 mM), naphthalene, phenanthrene, anthracene (2 and 10 mg per plate). Plates were incubated up to 28 days at 1 °C, 10 °C and 20 °C.

#### 2.9.8. Enzyme Activities

Amylase, protease, cellulase and esterase-lipase activities were tested with two replicates as described [54,55] on R2A agar supplemented with starch, skim milk (each compound 0.4% w/v), carboxymethylcellulose and trypan blue (0.4% and 0.01% w/v, respectively) or Tween 80 and CaCl_2_ (0.4% v/v and 0.01% w/v, respectively). Ligninolytic activity was evaluated on MM agar plates containing 0.4% (w/v) lignosulfonic acid sodium salt [56]. Plates were incubated up to 21 days at 1, 10 and 20 °C.

## 3. Results

### 3.1. Sediment Properties

The alluvial sediment material at the sampling site contained a mixture of pale sand, silt, clay and plant debris (litter). The predominant minerals were quartz and feldspar. Multiple stratifications occurred at intervals of 30–300 cm and contained dark-colored, sparsely silicified plant debris; sometimes fragments of stems of monocotyledoneus and dicotyledoneus trees and shrubs were visible. Fruits of conifers and walnut were found; walnut appeared in the sampling area in the warm period shortly before ice formation.

The investigated composite sediment sample had a dry mass content of 88%, a SOM content of 3.6% and a pH (CaCl_2_) of 4.5. Nutrient contents (nitrate, nitrite, phosphorus) were below the detection limit (<20 mg/kg dry sediment).

### 3.2. PLFA

The biomass estimate based on PLFA was 0.76 nmol g^−1^ dry sediment. Analysis of PLFA specific for bacteria, fungi and algae demonstrated the dominance of bacteria. Bacterial PLFA were detected to a (9.0 ± 1.2)-fold higher amount compared to fungi. Among bacterial PLFA, the analysis of fatty acids specific for Gram-positive and Gram-negative bacteria revealed a (1.8 ± 0.3)-fold higher amount of Gram-negative bacteria compared to Gram-positive bacteria. PLFA related to algae were not detected.

### 3.3. Direct Microbial Counts

Direct microbial counts after NPE revealed the presence of (4.7 ± 1.5) × 10^8^ cells g^−1^ sediment dry mass (mainly rods and occasionally fungal hyphae), which corresponded to approximately 0.02%–0.5% and 0.01%–0.6% of viable numbers obtained on R2A and 1/10 strength R2A agar, respectively.

When counting after staining with acridine orange, only green fluorescent cells that are often attributed to living cells, (2.5 ± 1.2) ×10^7^ cells g^−1^ sediment dry mass were counted after NPE (corresponding to 0.3%–9% of viable counts on R2A, see below), this number further increased after LE in the presence of nutrients and paralleled the increase in viable counts.

### 3.4. Enumeration of Culturable Bacteria and Fungi

**Figure 5:**
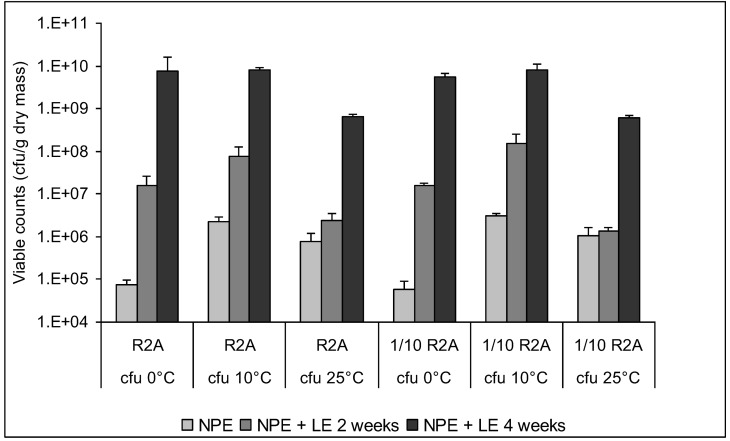
Effect of enrichment on numbers of culturable heterotrophic bacteria on R2A agar and 1/10 strength R2A agar incubated at 0, 10 and 25 °C. No growth was observed at 37 °C. **N**PE, natural permafrost enrichment (1 month at 0 °C in undisturbed sediment); **N**PE + LE 2 weeks, 2 weeks of liquid enrichment at 1 °C after NPE; **N**PE + LE 4 weeks, 4 weeks of liquid enrichment at 1 °C after NPE.

Independent of the enrichment period and of the culture medium, viable heterotrophic bacteria were found at 0 °C, 10 °C and 25 °C, but not at 37 °C (detection limit 100 cfu g^−1^ sediment) (Figure 5). However, the relation between bacteria able to grow at the different incubation temperatures was influenced by the period of enrichment. After NPE, viable bacterial numbers determined at 0 °C were 7.4 × 10^4^ (R2A agar) and 5.8 × 10^4^ g^−1^ sediment dry mass (1/10 strength R2A agar). They were 30-fold (R2A) or 50-fold (1/10 strength R2A) higher at 10 °C and 10-fold (R2A) or 18-fold (1/10 strength R2A) higher at 25 °C. Thus, only 3% and 10% of the viable bacterial numbers obtained on R2A and able to grow at 0 °C could also grow at 10 °C and 25 °C, respectively. An additional LE in the presence of nutrients after NPE resulted in an increase in viable numbers; this was also confirmed by counts of green fluorescent cells after staining with acridine orange. After two weeks of LE at 2 °C, the fraction of bacteria able to grow at 0 °C had increased to 20% of the fraction able to grow at 10 °C, but had decreased to 7% of the fraction able to grow at 25 °C. This trend was also observed after four weeks of LE when even 90% of the fraction able to grow at 10 °C could grow at 0 °C. An almost identical trend was observed for bacteria cultured on 1/10 strength R2A agar. Thus, enrichment in the presence of nutrients favored the enrichment of bacteria able to grow at 0 °C, while the opposite was observed for bacteria able to grow at 25 °C. Spore-forming bacteria were not detected at any of the tested incubation temperatures neither on R2A nor on 1/10 strength R2A agar (detection limit 100 cfu g^−1^ sediment).

In contrast to numbers of viable bacteria, numbers of viable fungi were very low and were only detected on media incubated at 0 and 10 °C, but not at higher incubation temperatures. After NPE, 2–5 fungal colonies appeared on all three media used to detect viable fungi. Colonies with the same appearance (color, size) also appeared after two and four weeks of LE in the presence of nutrients. The techniques applied in this study for the recovery of viable fungi might be limited since NPE was originally developed for bacteria, and subsequent LE might have favored bacteria rather than fungi.

Since these colonies did not differ in their visible appearance or growth behavior, only one of them was subjected to identification by CBS (Delft, The Netherlands) based on the rRNA gene sequence of the Internal Transcribed Spacer 1 and 2 (ITS). A sequence identity of 97% with a fungal endophyte associated with Antarctic mosses was detected. The strain was able to grow between 0 °C and 20 °C with fastest growth rates at 20 °C; sporulation was not detected.

### 3.5. Characterization of Culturable Bacterial Isolates

According to RFLP, the 32 analyzed bacterial isolates could be divided into three groups and the 16s rRNA gene sequence of two representatives of each restriction pattern were determined. 14 bacterial strains were identified as *Arthrobacter phenanthrenivorans* (98.1% sequence identity; GenBank accession number JX545208), 13 strains were identified as *Subtercola frigoramans* (99.4% 16S rDNA sequence identity; GenBank accession number JX545207), while only five strains could be affiliated to *Glaciimonas immobilis* (98.8% sequence identity; GenBank accession number JX545209). All *Arthrobacter phenanthrenivorans* and *Subtercola frigoramans* strains could be isolated after NPE and after additional LE. In contrast, none of the *Glaciimonas immobilis* strains were isolated after NPE, while all of them appeared after two weeks of LE, which indicates the selective enrichment of this species as already previously stated [37].

**Figure 6:**
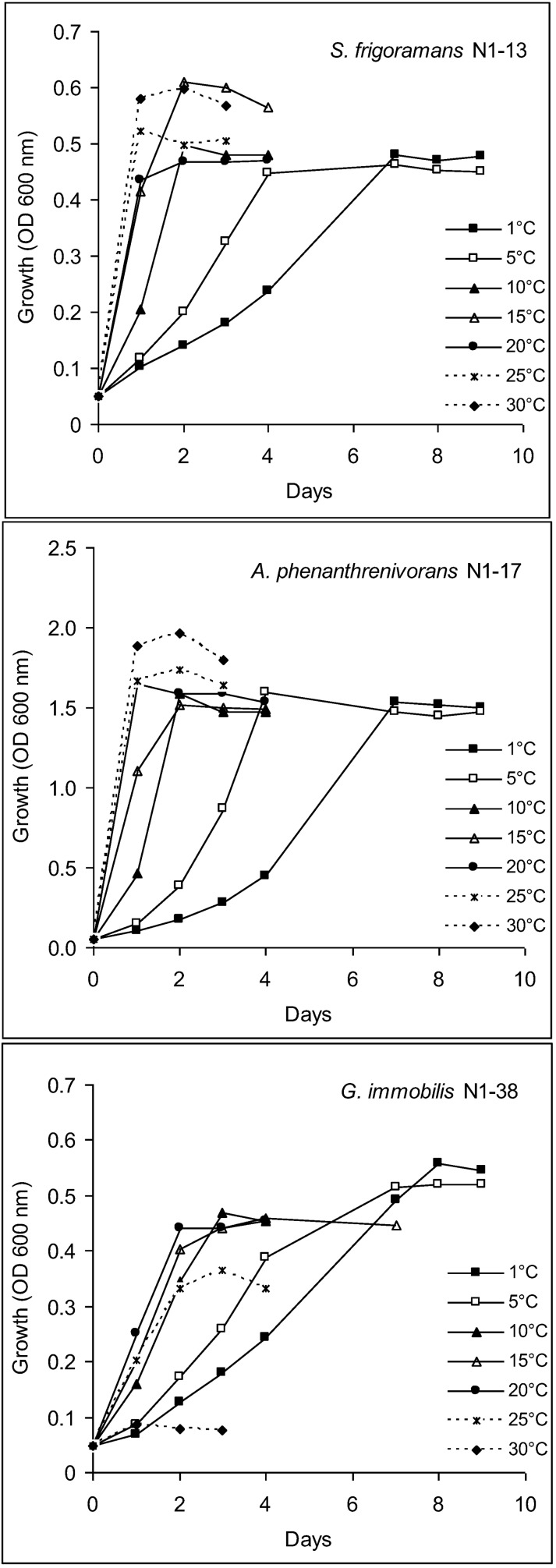
Effect of temperature on growth of bacterial strains isolated from ancient permafrost sediment (data at −5 °C were obtained without shaking and are therefore not shown).

Six strains (two representatives of each of the three identified bacterial species) were characterized with regard to their growth temperature range. Their ability to grow on different media, to produce enzymes, to grow in the presence of NaCl, antibiotics and heavy metals and to degrade hydrocarbons was evaluated at 1 °C, 10 °C and 20 °C. Characteristics of one representative for each bacterial species are shown in Table 1. All strains were able to grow at −5 °C, the upper temperature limit for growth in liquid culture was 30 °C for *Arthrobacter* and *Subtercola* strains, while *Glaciimonas* strains could grow up to 25 °C. All strains exhibited fastest growth rates in R2A broth at the maximum temperature for growth, biomass yield (cell density) was highest at 1–5 °C for *Glaciimonas*, while *Arthrobacter* and *Subtercola* strains produced the highest amount of biomass at 30 °C and their cell yields were *ca*. 20% lower at 1–25 °C than at 30 °C (Figure 6). *Arthrobacter* strains produced an approx. threefold higher amount of biomass than *Subtercola* and *Glaciimonas* strains. Growth at −5 °C is not shown in Figure 6 since the strains were cultured without shaking at this temperature.

All strains were initially exposed to oxygen and thus able to grow under aerobic conditions, but none of them was facultatively anaerobic. Growth on different media was temperature-dependent. All strains could grow at 1 °C, 10 °C and 20 °C on 1/10 strength R2A and R2A. *Arthrobacter* strains preferred rich media (NA, TSA, LB) at all three temperatures tested. *Subtercola* grew on NA at 10 °C and 20 °C but not at 1 °C, growth on LB was good at 20 °C, week at 10 °C and absent at 1 °C. *Glaciimonas* did not grow on rich media (NA, LB and TSA).

**Table 1:** Properties of bacterial strains isolated from ancient permafrost sediment.

Strain properties	*Subtercola frigoramans* N1-13	*Arthrobacter phenanthrenivorans* N1-17	*Glaciimonas immobilis* N1-38
**Tmin/Tmax**			
R2A broth	−5 °C/30 °C	−5 °C/30 °C	−5 °C/25 °C
R2A agar	1 °C/30 °C	1 °C/30 °C	1 °C/20 °C
**Growth on various media (R2A, NA, LB, TSA)**			
1 °C	R2A	R2A NA LB TSA	R2A
10 °C	R2A NA	R2A NA LB TSA	R2A
20 °C	R2A NA LB	R2A NA LB TSA	R2A
**Growth in presence of NaCl (% w/v)**			
1 °C	0%	1% weak	0%
10 °C	0% (1% weak)	2%	0%
20 °C	1%	3% (5% weak)	0%
Growth in presence of cyclosporin A (100 µg mL^−1^) *			
1 °C	Weak	+	weak
10 °C	+	+	+
20 °C	+	+	+
**Utilization of lignosulfonic acid**			
1 °C	−	+	−
10 °C	Weak	+	−
20 °C	Weak	+	−
Resistance to heavy metals (0.1 mM Cu^2+^, 1 mM Pb^2+^) **			
1 °C	−	−	−
10 °C	(+)	+	−
20 °C	+	++	−

* All strains were sensitive to rifampicin, kanamycin, tetracyclin, streptomycin, chloramphenicol (20 µg mL^−1^ and 100 µg mL^−1^) at 1 °C, 10 °C and 20 °C. ** All strains were sensitive to 1–5 mM Zn^2+^, 3–5 mM Pb^2+^ and 1–3 mM Cu^2+^ at 1 °C, 10 °C and 20 °C.

*Arthrobacter* exhibited a higher salt tolerance than all other strains. Generally, an increased sensitivity to NaCl was noted at decreased temperatures. All strains could grow in the presence of cyclosporin A and at 10 °C and 20 °C, growth at 1 °C in the presence of this antibiotic was weak for *Subtercola*. *Arthrobacter* strains showed weak resistance towards low amounts (20 µg mL^−1^) of penicillin and ampicillin at 20 °C but behaved sensitive at 10 °C and 1 °C. None of the strains could grow in the presence of other antibiotics tested in this study (rifampicin, kanamycin, tetracyclin, streptomycin, chloramphenicol; 20 or 100 µg mL^−1^) at 1 °C, 10 °C or 20 °C on agar plates. However, strain *Arthrobacter phenanthrenivorans* N1-17 showed growth in the presence of a number of antibiotics in liquid culture, whereby resistance was clearly influenced by the growth temperature and was generally highest at 20 °C and lowest at 1 °C (Table 2). All tested strains in this study were sensitive to 1–3 mM Cu^2+^ and 1–3 mM Zn^2+^. *Subtercola* and *Arthrobacter* strains were resistant to 0.1 mM Cu^2+^ and to 1 mM Pb^2+^ at 10 °C and 20 °C, with a more pronounced resistance at 20 °C than at 10 °C.

**Table 2:** Effect of temperature on sensitivity of *Arthrobacter phenanthrenivorans* N1-17 to antibiotics in R2A broth.

Antibiotic (20 µg mL^−1^)	Relative growth (%)		
	1 °C	10 °C	20 °C
Without antibiotic	100	100	100
Chloramphenicol	24	36	45
Kanamycin	28	41	52
Rifampicin	25	38	46
Streptomycin	26	36	50
Tetracyclin	28	37	48

None of the strains produced protease, amylase, lipase (Tween 80 hydrolysis) or CM-cellulase. Ligninolytic activity was noted for *Arthrobacter* at 10 °C and 20 °C and was weak at 1 °C. Since the substrate used for this activity test, lignosulfonic acid, contains a high amount of phenolic compounds, these strains were expected to degrade phenol [56], however, none of the strains was able to utilize n-hexadecane, diesel oil, phenol, naphthalene, phenanthrene or anthracene at any of the temperatures tested.

## 4. Discussion

Microbial abundance in permafrost varies depending on the environment. Microbial permafrost communities contain culturable, viable-but-non-culturable, non-culturable and dead cells [57]. Due to constant subzero temperatures in permafrost, dead or compromised microbial cells may remain well preserved and contribute to total microbial counts [6]. The presence of partially degraded bacterial cells and empty “ghost” cells has been demonstrated in Siberian permafrost [58]. Siberian permafrost is dominated by very small (≤1 µm) cells or ultramicroforms of cells (≤0.4 µm) [57], which are typical of the viable but non-culturable state [59]. The microscopic investigation in this study also revealed the dominance of small-sized cells.

Data from direct and microbial counts reported in this study are within the range of data described in other studies on permafrost. Viable counts of aerobic heterotrophs in Siberian permafrost range from 0 to 10^8^ cfu g^−1^ material [6,37,60]. Viable counts obtained in our study after NPE were (6–7) × 10^4^ cfu g^−1^ dry sediment. Direct counts by epifluorescence microscopy, which is frequently used for the enumeration of bacteria in environmental samples [61,62], in Siberian permafrost range from 10^3^–10^8^ cells g^−1^ investigated material [5,6,57,60]; ancient Siberian permafrost sediments (100,000 years old, from late Pleistocene) contained 2 × 10^7^ to 1.2 × 10^8^ cells g^−1^ sediment [37]. The percentage of viable counts in relation to direct microscopic counts (DTAF staining) ranged from 0.02% [63] to <0.01%–0.3% [37]. Higher fractions of viable cells (0.1%–10%) were counted with acridine orange staining [39]; we obtained very similar values (0.3%–9% of acridine orange-stained cells were culturable after NPE) in our study, these values are also in agreement with others [64] who reported that 1%–10% of cells stained with acridine orange are culturable.

The successful recovery of viable cells from permafrost depends on a number of factors. The occurrence of viable microorganisms was independent of the depth of permafrost sampling and sometimes even increased with depth [65]. The number of bacterial isolates has been reported to decrease with increasing permafrost age, while species diversity remained almost unaffected [65]. Viable microbial cells could be recovered from 3-million-year-old Siberian permafrost [7,11,66]. Long-term survival of bacteria in 500,000-year-old permafrost samples was closely tied to cellular metabolic activity and DNA repair that, over time, may be superior to dormancy as a strategy to sustain viability [3].

Other important factors for the successful recovery of permafrost microorganisms are low-temperature enrichment strategies and media composition. NPE of unthawed (undisturbed) permafrost soil at 4 °C for up to 12 weeks resulted in enhanced recovery of permafrost bacteria and led to the isolation of genotypes that could not be recovered by means of low-temperature liquid enrichments, since diverse soil microbial communities can better develop independently in various soil microenvironments than in liquid culture [37]. Therefore, we applied this enrichment strategy in our study. We additionally enriched permafrost microorganisms after NPE in LE in the presence of nutrients, which resulted in increased viable counts and in the isolation of representatives of the genus *Glaciimonas* that could not be isolated after NPE, while all representatives of *Arthrobacter phenanthrenivorans* and *Subtercola frigoramans* could be isolated after NPE and after additional LE.

Rich media favor morphological diversity, while diluted media (with low nutrient contents) enhance the quantitative recovery of viable microorganisms [37]. In our study, we did not observe significant differences between viable counts in R2A and 1/10 strength R2A medium, which demonstrates that R2A is a suitable medium for the isolation of oligotrophic microorganisms from environmental habitats such as permafrost.

Both Gram-positive and Gram-negative bacteria have been described in Siberian and other permafrost samples. *Firmicutes* and *Actinobacteria* generally represent a high proportion of the bacterial permafrost community and accounted for 45% of Siberian isolates [67]; *Arthrobacter* (*Actinobacteria*) and *Planococcus* (*Firmicutes*) accounted for 85% of cultured isolates from a northeast Siberian permafrost sample [68]. In our study, the majority of the identified bacterial isolates could be attributed to *Actinobacteria*: *Arthrobacter phenanthrenivorans*, previously isolated from creosote oil-polluted soil [69], and *Subtercola frigoramans*, so far only found in cold groundwater [70]. Only a small fraction belonged to the species *Glaciimonas immobilis* (*Betaproteobacteria*) previously found in alpine glacier cryoconite [71]. PLFA analysis (a culture-independent approach), however, demonstrated an approximately two-fold higher amount of Gram-negative compared to Gram-positive bacteria. Unfortunately no PLFA data are available for permafrost, which makes it impossible to compare our values. Microbial community analysis of subalpine and alpine soils demonstrated a general decrease of PLFA representing bacteria and fungi, as well as a shift of the bacterial population towards the increase of the Gram-negative population with altitude [40].

The high percentage of high G + C Gram-positive, non-spore-forming bacteria (such as *Actinobacteria*) among ancient permafrost isolates has been attributed to their adaptation to frozen environments, to their metabolic activity at low temperatures, to their ability to form dormant cells, to their efficient DNA repair mechanisms, and to the fact that they are more easily cultured [3,66,68]. In contrast, the dominance of spore-forming bacterial genera in Canadian permafrost was attributed to the ability of this microbial group to survive as spores rather than vegetative cells [63]. The first metagenomic analysis of permafrost samples confirmed that *Actinobacteria* are well adapted to the conditions prevailing in permafrost habitats [72].

The abundance of spore-forming bacteria varies between geographically isolated permafrost samples [5,6]. In Siberian permafrost samples, they represented 1%–30% of viable isolates [67] or were not at all detected [68]. This was also the case in our study. However, Brushkov *et al*. [11] isolated *Bacillus* sp. strains from 3-million-year-old permafrost.

There is only little information on fungi in permafrost habitats. Viable fungi can be isolated from permafrost, however, fungal abundance is low, while species diversity is high [73].

Permafrost microorganisms are primarily cold-adapted and only few representatives are mesophilic or thermophilic [4,6,7]. Growth at 37 °C has been rarely reported [68]. Steven *et al*. [63] reported a three-fold higher amount of viable cells growing at 5 °C compared to viable counts at 25 °C; in our study, only 10% of culturable cells growing at 0 °C could also grow at 25 °C. A number of cold-adapted permafrost microorganisms are able to grow at subzero temperatures down to −10 °C [4,63,66,68]. In our study, all strains investigated could grow at −5 °C, and the maximum temperature for growth ranged from 20 to 30 °C.

Permafrost microorganisms tend to be more halotolerant than organisms from the overlaying active layer [6,74], which is seen as a microbial survival strategy in environments with low water activity, such as permafrost, where little water is bioavailable [75]. Tolerance to 7% NaCl [63] or to 8% NaCl [68] has been reported. *Arthrobacter* strains in our study tolerated up to 3% NaCl and showed weak growth in the presence of 5% (w/v) NaCl at 20 °C; however, sensitivity to salt increased when temperature decreased, which has not yet been described before.

Permafrost bacteria are resistant to a wide range of antibiotics combined with the presence of mobile genetic elements [76], which might be part of a generalized bacterial response to stress conditions [77]. Metagenomic analysis of ancient DNA from 30,000-year-old Beringian permafrost sediments demonstrated the presence of a highly diverse collection of genes encoding resistance to ß-lactam, tetracycline and glycopeptide antibiotics [78]. The strains investigated in our study were resistant to cyclosporin A (an immunosuppressive cyclopeptide) and sensitivity towards this compound increased when temperature decreased. The same tendency was noted when growth was tested in the presence of other antibiotics in liquid culture. Similarly, a trend of increased antibiotic sensitivity at 4 °C *versus* 24 °C with all classes of antibiotics except erythromycin was described for permafrost bacteria [66,79].

## 5. Conclusion

In conclusion, our data demonstrate the presence of viable bacteria in ancient Siberian permafrost. The sample was collected from an ancient Neogene deposit that was permanently frozen for 3.5 million years. The low diversity of viable microorganisms observed in the studied sample may be attributed to a number of factors, such as strong selection pressure due to harsh conditions, nutrient deficiency, presence of inorganic and organic inhibitors, permanently freezing conditions for 3.5 million years combined with the lack of contamination by percolating water from surface, groundwater, lakes and rivers. Since this is the first study of permafrost microbial diversity in the area of Central Yakutia, we cannot compare our data with those of others. Bacterial isolates were able to grow at subzero temperatures and some were halotolerant. In spite of the ligninolytic activity of some strains, no biodegradation activity was detected. In general, sensitivity to rich media, antibiotics, heavy metals, and salt increased when temperature decreased (20 °C > 10 °C > 1 °C). This could be explained as the reaction to an increased stress situation at low temperatures. However, further studies are needed to elucidate the mechanisms behind this process.
